# Pharmacological considerations for next-generation protein therapeutics in cardiovascular disease

**DOI:** 10.1016/j.jpet.2025.103696

**Published:** 2025-12-30

**Authors:** Emily Lin, Noor Momin

**Affiliations:** 1 Department of Bioengineering, University of Pennsylvania, Philadelphia, Pennsylvania; 2 Center for Precision Engineering for Health, University of Pennsylvania, Philadelphia, Pennsylvania; 3 Cardiovascular Institute, Perelman School of Medicine, University of Pennsylvania, Philadelphia, Pennsylvania; 4 Institute for Immunology, Perelman School of Medicine, University of Pennsylvania, Philadelphia, Pennsylvania; 5 Institute for Regenerative Medicine, Perelman School of Medicine, University of Pennsylvania, Philadelphia, Pennsylvania

**Keywords:** Pharmacokinetics, Pharmacodynamics, Cardiovascular disease, Protein engineering, Antibodies, Multispecific proteins

## Abstract

Cardiovascular disease (CVD) remains the leading cause of death worldwide despite decades of therapeutic advances. Emerging insights into its etiology have revealed previously unappreciated cellular and molecular drivers beyond traditional risk factors, prompting the development of treatments that target newly identified culprit proteins and cells within cardiovascular tissues. Protein-based biologics—particularly monoclonal antibodies and multispecific proteins—are known for their strength and specificity in targeting and their established use as treatments for other diseases. However, extending biologics to new indications faces challenges: achieving durable effects in diseased tissues and minimizing side effects in healthy tissue. Addressing these long-standing challenges requires fine-tuning biologics’ pharmacokinetic properties and pharmacodynamic effects according to target- and disease-specific requirements. In this review, we examine foundational pharmacokinetic and pharmacodynamic principles in the context of cardiovascular-targeted biologics, highlighting the role of protein design in controlling distribution, efficacy, and safety. Additionally, we discuss emerging preclinical and clinical biologics specifically designed for CVDs, as well as emerging opportunities in this landscape. These advances point toward a future where pharmacokinetics guide the rational design of next-generation protein therapeutics for CVD.

**Significance Statement:**

Protein-based biologics hold promise for treating cardiovascular diseases (CVD); however, their successful translation requires understanding how proteins’ properties and cardiovascular physiology shape pharmacokinetic and pharmacodynamic behavior. This minireview connects foundational pharmacology principles with strategies in protein engineering suitable for CVD applications. Pharmacokinetic-guided design will accelerate the development of protein therapies that can transform CVD treatment.

## Graphical abstract

**Figure:**
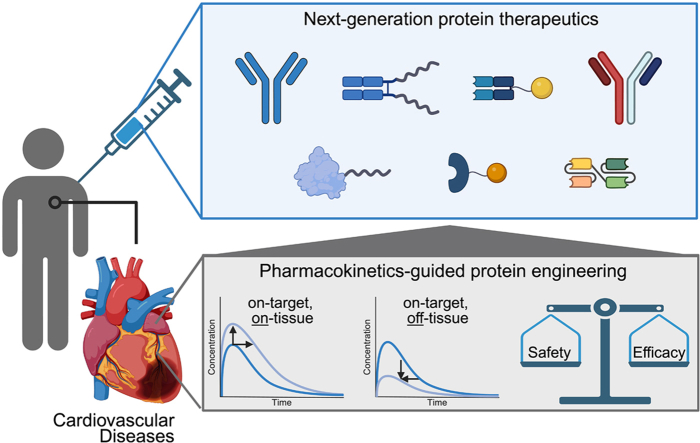

## 1 Introduction

Cardiovascular disease (CVD) is a global health crisis. It affects over 520 million people and accounts for nearly one-third of deaths worldwide.^1^, ^2^, ^3^ Given the prevalence and severity of CVD, there is an urgent clinical need for new treatments. CVD describes a panoply of disorders that afflict the heart and blood vessels. The most prevalent and lethal conditions include coronary and peripheral artery disease, heart failure, and arrhythmia.^1^ Many risk factors that fuel the development of these diseases are well recognized. For example, persistent high blood pressure or hypertension, elevated cholesterol, obesity, and age progressively strain the cardiovascular system.^4^ Thus medications such as aspirin, statins, *β*-blockers, angiotensin-converting enzyme inhibitors, angiotensin II receptor blockers, and mineralocorticoid receptor antagonists, which are aimed at stalling these recognized risk factors, have revolutionized CVD prevention and treatment.^5^, ^6^, ^7^, ^8^ But despite these highly effective therapies, CVD continues to impose a substantial and growing burden. Uncovering additional pathophysiologic drivers—and engineering a new toolkit to target them—represents the next frontier in cardiovascular medicine.

The cardiovascular system relies on multiple specialized, intricately organized cell types. The heart itself is comprised of cardiomyocytes—which are the main facilitators of its forceful contraction—however, recent single-cell analysis shows that over 50% of total cells in a healthy human myocardium are actually fibroblasts, mural, endothelial, and immune cells.^9^ Similarly, healthy vessels contain diverse arrangements of endothelial, mural, immune cells, and fibroblasts.^10^ In settings of CVD, the afflicted heart and/or vessels undergo dramatic shifts in their cell populations’ abundance and phenotype.^9^^,^^11^, ^12^, ^13^, ^14^, ^15^, ^16^, ^17^ Recent and ongoing efforts have begun to unearth how specific proteins, cells, and signaling pathways relate to, and in some circumstances even drive, disease. These discoveries now pave clear and promising paths toward targeted treatment.^18^ However, the challenge still lies in modulating these target proteins, cells, and signaling pathways both specifically and durably. To this end, many modalities are being investigated. Biologics represent a distinct class of clinically validated drugs that offer both the precision and persistence needed for a safe and effective therapy.

Biologics describe many types of drugs derived from living sources. This review focuses on *protein-*based biologics, specifically monoclonal antibodies and multispecific proteins. These highly versatile modalities offer unique opportunities to modulate proteins and cells identified as targets of CVD. Recent advances in the field of protein engineering have even accelerated their development. However, the successful translation of biologics into therapeutics in a CVD context hinges on the careful optimization of their pharmacokinetics and pharmacodynamics.^19^

Pharmacokinetics (PK) describes how the body processes a drug over time. PK processes include: absorption into the bloodstream from the site of administration, distribution throughout various tissues and organs, metabolism into broken down metabolites, and excretion from the body—collectively referred to as ADME. The properties of a biologic (size, charge, structure, and target binding), its interactions with other biomolecules, cells, tissues, and organs in the body, and the variability across patients all collectively influence ADME and thus PK. Given this tremendous complexity, accurately predicting the PK of drugs remains a significant challenge. Therefore, it is essential to consider how specific design choices in protein engineering generally impact PK, particularly in the context of the cardiovascular system.

Pharmacodynamics (PD) describes how a drug affects the body. PK and PD are deeply interconnected: the persistence and distribution of a biologic in the body directly influences its ability to engage its target(s), elicit an effect, and ultimately yield a favorable safety and efficacy profile. As such, optimizing PK is a powerful lever for fine-tuning PD. This relationship is particularly relevant for protein-based biologics, which can be easily engineered. In this review, we first focus on monoclonal antibodies—the most well characterized protein biologics—as exemplars of generalizable PK/PD principles, then discuss how these principles can be applied to more complex multispecific CVD therapies. These insights are crucial in ushering in a next-generation of safe and effective protein-based biologics for CVD treatment.

## 2 Monoclonal antibodies illustrate protein PK/PD principles

### 2.1 Overview

In 1986, Muromonab-CD3 (OKT3)—a monoclonal antibody (mAb) targeting CD3 on T cells to prevent organ transplant rejection—became the first mAb approved by the Food and Drug Administration (FDA).^20^ Since then, over a hundred mAbs have earned FDA approval, making them the fastest growing class of medications in the United States. These antibodies now serve as mainstays in treating various diseases, including cancers (pembrolizumab and nivolumab) and autoimmune diseases (adalimumab and ustekinumab).^21^^,^^22^ Given their clinical dominance, mAbs represent the most extensively studied biologic modality, and their PK/PD principles are among the best characterized. Here, we use mAbs to provide a foundational framework for understanding general PK/PD principles that can be applied across other protein-based biologics.

Therapeutic mAbs resemble the body’s natural antibodies. They consist of 2 main regions: the fragment antigen-binding (Fab) region contains the variable domains that bind specific targets, and the fragment crystallizable (Fc) region determines the antibody’s effector functions and half-life. As therapeutics, mAbs’ Fab region can be customized to specifically bind any target molecule including extracellular or cell surface proteins; binding can in turn stimulate, inhibit, or alter the disposition of a target protein. The Fc region can also be customized to either recruit immune effector functions—such as complement, antibody-dependent cellular cytotoxicity, or phagocytosis—or simply provide half-life extension without effector activity.^23^, ^24^, ^25^ This precision in recognizing specific epitopes and versatility in modulating biological pathways makes mAbs an attractive therapeutic option for CVD.

CVD is aggravated by complex pathophysiology, including dysregulated metabolism, chronic inflammation, and tissue fibrosis. mAbs have been developed to address each of these processes. For metabolic dysfunction, mAbs can reduce serum low-density lipoprotein (LDL) cholesterol levels—alirocumab and evolocumab both inhibit proprotein convertase subtilisin/kexin type 9 (PCSK9), which enhances LDL receptor recycling.^26^ For inflammation, mAbs can target key inflammatory mediators—canakinumab inhibits interleukin-1*β* (IL-1*β*) and mepolizumab inhibits IL-5.^27^^,^^28^ For tissue remodeling and fibrosis, mAbs can target cells and pathways that drive pathological cardiac remodeling—MLN1202 inhibits C-C chemokine receptor type 2, a chemokine receptor that recruits inflammatory monocytes to sites of cardiac injury, and several preclinical antibodies inhibit platelet-derived growth factor receptor signaling, which promotes fibroblast proliferation and collagen deposition.^29^, ^30^, ^31^, ^32^ These examples highlight the proven utility of mAbs across diverse pathophysiologic processes relevant to CVD. When applying existing therapies or engineering new mAbs for cardiovascular indications, it is crucial to consider how their PK could ultimately influence their efficacy and safety profiles as therapeutics.

### 2.2 Pharmacokinetic determinants

The PK properties of mAbs are governed by multiple mechanisms. These include nonspecific proteolysis and cellular catabolism, neonatal Fc receptor (FcRn)-mediated recycling, target-mediated drug disposition (TMDD), and potential immunogenicity. The relative importance of each process depends on the antibody, its target, and the patient.

**Fig. 1:**
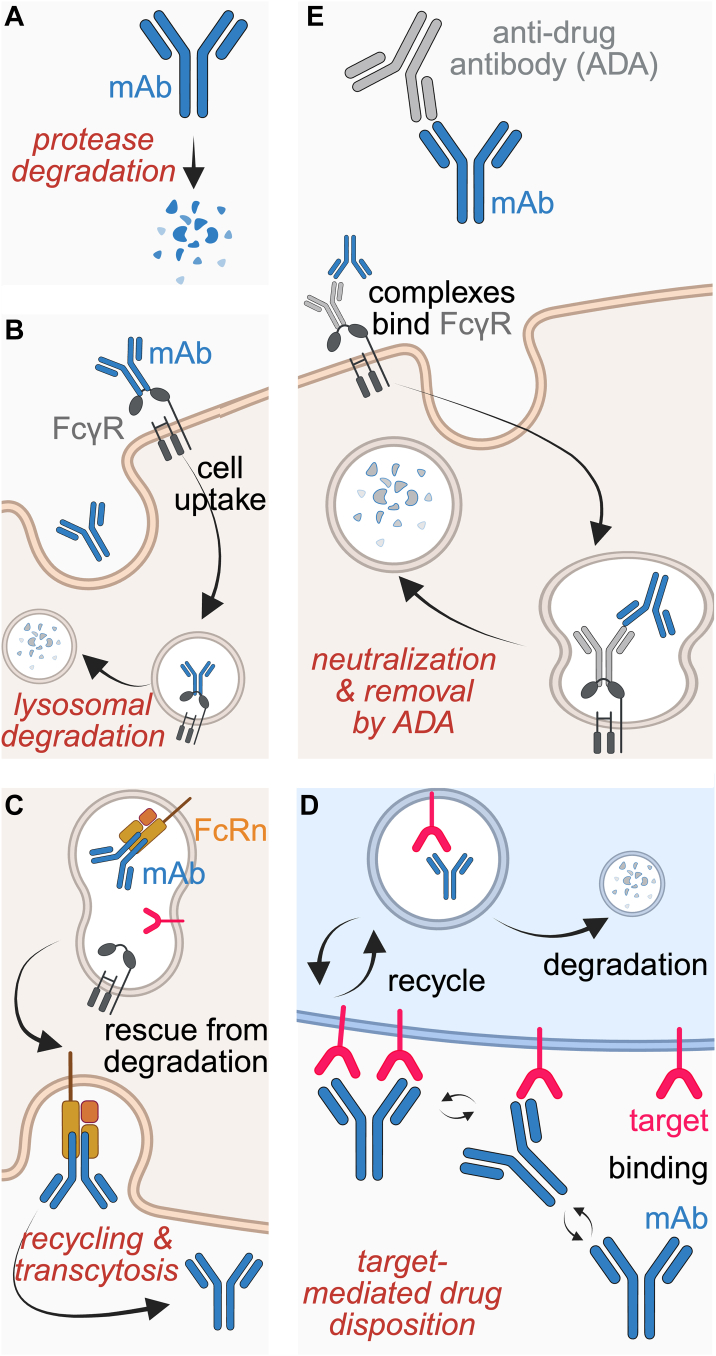
Pharmacokinetic considerations for monoclonal antibodies. (A) Nonspecific protease-mediated degradation in circulation. (B) Fc *γ* receptor (Fc*γ*R)-mediated cellular uptake and lysosomal degradation. (C) FcRn-mediated recycling or transcytosis, rescuing mAbs from degradation and extending half-life. (D) TMDD, where target binding can lead to pharmacokinetic changes. (E) Antidrug antibody (ADA) formation can neutralize mAb activity or complex to promote Fc*γ*R-mediated clearance. Created in BioRender (2025), https://BioRender.com/kyxrle6.

mAbs are large proteins (∼150 kDa) that exceed the molecular weight cutoff for renal filtration and therefore are not typically excreted in the urine.^33^^,^^34^ Instead, they are subjected to degradation and clearance in other ways. Proteases in the blood or extracellular tissue environment can cleave antibodies, often at the hinge region connecting the Fab and Fc regions (Fig. 1A). Phagocytic cells, primarily in the liver, can remove antibodies via binding to the Fc region.^35^ Once internalized by these cells, mAbs are trafficked to lysosomes where they undergo degradation into amino acids and peptides (Fig. 1B). These breakdown products are then recycled or eliminated via hepatic metabolism.^36^^,^^37^ Meanwhile, nonspecific cellular uptake can occur across all tissues via pinocytosis or nonreceptor-mediated uptake, leading to slower degradation independent of target or Fc receptor engagement.^38^

FcRn-mediated recycling fundamentally alters this clearance pattern by rescuing antibodies from degradation within a cell. When mAbs are internalized into cells, FcRn present in various cell types can bind the Fc domain with high affinity in acidic endosomes (pH ∼6.0). The antibody-FcRn complex then traffics back to the cell surface, where it dissociates at physiological pH (∼7.4), returning the intact antibody to circulation or the tissue interstitium (Fig. 1C).^36^^,^^37^ This pH-dependent salvage mechanism enables mAbs to achieve extended half-lives of 11 to 30 days.^36^^,^^37^^,^^39^ For example, evolocumab—a fully human IgG2 antibody targeting PCSK9 used to treat hyperlipidemia and reduce the risk of a myocardial infarction—exhibits a half-life of 11 to 17 days, largely attributable to FcRn-mediated protection.^40^^,^^41^ Although this extended circulatory half-life that enhances mAb exposure to the target in disease tissues is correlated with effectiveness, it also prolongs the likelihood of unintended interactions within healthy tissues, potentially leading to adverse effects, discussed later.

The therapeutic target itself is another determinant of mAb PK.^36^ TMDD occurs when an antibody’s binding to its intended target significantly shifts its PK. This process is especially pertinent with cellular targets. When an antibody binds to its target protein on a cell, the antibody-target complex is often internalized and degraded, effectively removing it from circulation (Fig. 1D). At low antibody doses, clearance may be dominated by rapid target binding and internalization. As the dose increases and targets become saturated, clearance shifts toward slower, nonsaturable, and nonspecific degradation, causing a nonlinear exposure-dose relationship.^36^^,^^42^^,^^43^ The magnitude of TMDD depends on both target expression levels in the patient and the binding kinetics of the engineered antibody. Affinity plays a central role—high-affinity mAbs are more likely to undergo rapid, saturable clearance via target binding, leading to pronounced nonlinearity in PK. Lower-affinity mAbs may exhibit reduced target-mediated clearance; however, this weak binding may compromise efficacy if target engagement becomes insufficient.^36^^,^^43^^,^^44^ Therefore, optimizing affinity is critical to balance efficacy with desirable PK properties. The clinical relevance of TMDD is demonstrated by alirocumab, an FDA-approved PCSK9-targeting mAb.^43^^,^^45^^,^^46^ Alirocumab exhibits TMDD characterized by rapid, nonlinear clearance at low concentrations and slower, linear clearance once PCSK9 becomes saturated. Population PK modeling using Michaelis-Menten kinetics accurately described this dual-phase clearance and quantified the impact of covariates such as statin use, which increases PCSK9 levels and shifts the balance toward target-mediated clearance.^43^ This example highlights how mechanistic understanding of TMDD is essential for guiding mAb development and clinical application in CVD.

Finally, antidrug antibodies (ADAs) contribute to interpatient variability observed in the PK of both CVD and non-CVD biologics. ADAs are produced by a patient’s immune system in response to an exogenous biologic. These ADAs can impair a treatment’s effectiveness and alter its PK.^47^ ADAs can neutralize the biological activity of therapeutic mAbs directly by blocking its Fab’s binding, or indirectly by forming immune complexes that accelerate clearance from circulation, reducing the drug’s exposure and therapeutic impact (Fig. 1E). The presence and formation of ADAs depend on the mAb’s sequence, structural features (eg, glycosylation), aggregation, cell of origin, and impurities. ADA formation can also vary widely across patients owing to patient-specific variables such as immune and human leukocyte antigen status.^48^^,^^49^ Repeated or long-term dosing can heighten the likelihood of ADA formation, because each exposure gives the immune system further opportunity to recognize the biologic as “foreign.”^49^ An instance in CVD therapy is the humanized anti-PCSK9 antibody bococizumab, which induced high-titer ADAs in 48% of patients and neutralizing antibodies in 29% of patients in the Studies of PCSK9 Inhibition and the Reduction of Vascular Events trials, with many of these responses emerging only after 12 weeks dosing every 2 weeks. These ADAs titer-dependently attenuated the LDL-lowering effect and contributed to wide variability in treatment outcomes (−42.5% change in LDL cholesterol level for patients without detectable ADAs compared with −12.3% for patients with ≥1:5674 ADA titer ratio), ultimately contributing to discontinuation of its trial in 2016.^50^^,^^51^ In contrast, fully human PCSK9 inhibitors such as alirocumab and evolocumab have demonstrated far lower immunogenicity in comparable patient populations. ADAs were detected in 5.1% of patients treated with alirocumab and 0.1% of patients treated with evolocumab), supporting the importance of sequence humanization (bococizumab sequence is ∼3% murine).^41^^,^^52^, ^53^, ^54^ Nonetheless, even fully human antibodies are not exempt from ADAs, particularly in settings of repeated dosing or heightened inflammation. As such, engineering strategies such as epitope deimmunization (removal of T cell epitopes), reduction of aggregation, and optimized glycosylation are commonly used to lower ADA risk.^55^, ^56^, ^57^, ^58^ In silico epitope mapping, major histocompatibility complex-binding prediction, and dendritic cell loading assays are more recent strategies to help identify high-risk candidates and guide the design of mAbs with lower immunogenicity profiles.^59^, ^60^, ^61^, ^62^, ^63^, ^64^, ^65^ Using these tools alongside a deeper understanding of the mechanisms that drive immunogenicity enables rational design of mAb therapeutics. In the case of bococizumab, a modified variant incorporating aggregation-reducing mutations demonstrated markedly reduced immunogenicity in preclinical models, validating how design improvements informed by ADA profiling can reduce immunogenicity risk.^66^ For mAbs intended to treat CVD, which may require chronic administration, minimizing immunogenicity is especially critical to preserve PK, efficacy, and safety across diverse patient populations.

### 2.3 Pharmacodynamics

The high specificity and durability of mAbs offers significant advantages, allowing them to precisely modulate disease-relevant pathways. However, many therapeutic targets—such as receptors, ligands, or matrix components—are not exclusive to diseased tissue but are instead only overexpressed relative to healthy tissue.^67^^,^^68^ As a result, mAbs can still bind their targets that are present in nondiseased areas (ie, on-target and off-tissue activity), leading to potentially adverse side effects.^69^^,^^70^ To achieve additional tissue specificity and minimize toxicity, there have been efforts to design mAbs that preferentially engage targets that are exclusively upregulated in diseased (infarcted, fibrotic, or inflamed) tissue, or to carefully optimize dose, schedule, and/or administration route.

mAbs can precisely bind targets involved in CVD, as demonstrated by many examples. Evolocumab and alirocumab, mentioned previously, target PCSK9. PCSK9 is predominantly expressed in the liver—its intended target tissue—and is upregulated in disease states, and thus is largely devoid of on-target, off-tissue side effects.^71^ However, many promising targets present greater challenges because of their broader off-tissue distribution. IL-1*β* is a proinflammatory cytokine that is elevated in atherosclerotic plaques, prompting the evaluation of canakinumab—an FDA-approved biologic that targets IL-1*β*—to treat atherothrombosis in the Canakinumab Anti-Inflammatory Thrombosis Outcomes Study trial. However, unlike PCSK9 whose expression is largely restricted to the liver, IL-1*β* is found throughout the body and is essential for innate immune responses. Thus, treatment with canakinumab led to systemic disruption of the body’s inflammatory response that led to a significant increase in fatal infection and sepsis.^72^ Similarly, CD40, a costimulatory receptor on B cells involved in adaptive immune activation, is upregulated in atherosclerotic plaques but is also expressed in other tissues such as the endothelium and other immune cells. Antagonizing B cells’ CD40 interaction with CD154 on T cells using mAbs has shown promise in treating atherosclerosis and inhibiting transplantation rejection in preclinical models.^73^, ^74^, ^75^, ^76^ However, anti-CD40 and anti-CD154 mAbs have previously been associated with thromboembolic complications in clinical trials—due to binding of CD154 on off-target platelets triggering Fc *γ* receptor crosslinking resulting in platelet aggregation—but those concerns are currently being addressed with mAb engineering to prevent platelet activation.^77^, ^78^, ^79^, ^80^ Anti-CD47 mAbs, which were originally developed for oncology, have also been explored to treat CVD.^81^^,^^82^ In atherosclerosis, CD47 is upregulated in diseased vascular tissue, where it impairs clearance of apoptotic cells, and CD47-blocking mAbs have shown efficacy in restoring phagocytic clearance and reducing plaque burden in preclinical models.^83^ However, their ubiquitous expression of CD47—particularly its high expression on red blood cells and platelets—creates a substantial antigen sink that necessitates high doses of therapeutic antibodies in order to achieve sufficient on-tissue targeting, thereby increasing the risk of off-tissue anemia and thrombocytopenia.^84^, ^85^, ^86^ One approach that has been explored to mitigate these on-target, off-tissue effects in oncology settings has been the development of agents such as evorpacept (ALX148), a CD47-blocking biologic with an inactive Fc domain that avoids engaging Fc *γ* receptor on macrophages, thereby reducing off-target cytotoxicity and improving tolerability in clinical trials.^87^, ^88^, ^89^, ^90^, ^91^

However, the heart is a common off-target organ in oncology treatments, such as programmed cell death protein 1 (nivolumab and pembrolizumab), programmed cell death ligand 1 (avelumab and atezolizumab), and cytotoxic T-lymphocyte-associated protein 4 (ipilimumab) inhibitors.^92^ Although these mAbs have shown remarkable clinical response in various cancers, these targets are broadly expressed on all T cells to prevent them from attacking healthy tissues, and systemic blockade of these regulators can lead to the immune system attacking healthy heart tissue and immune-related adverse events, including myocarditis.^93^, ^94^, ^95^, ^96^, ^97^ The heart, in particular, is an organ with tightly regulated electrophysiological and structural function, such that inappropriate immune activation—due to immune checkpoint blockades or ADAs formed in response to biologics—can lead to especially severe consequences, including autoimmune myocarditis, exacerbation of atherosclerotic plaques, or conduction abnormalities such as heart block.^97^^,^^98^ This sensitivity to minor perturbations emphasizes the need for achieving superior on-target, on-tissue specificity, particularly when considering the heart.

## 3 Multispecific proteins using PK principles to tune PD

### 3.1 Overview

The extensive development and clinical use of mAbs have not only demonstrated their therapeutic potential but also provided a rich foundation for understanding how their features generally influence the PK/PD of protein-based biologics. Many of the PK principles elucidated in the context of mAbs—such as half-life extension via Fc engineering, receptor-mediated targeting and endocytosis, and tissue distribution—can be strategically leveraged and expanded upon in the design of next-generation multispecific therapeutics.

Multispecific proteins combine ≥2 protein domains into a single therapeutic construct. This format can lend improvements to PK that enhance the efficacy and safety of biologics beyond what monoclonal antibodies or single-domain proteins can provide alone. Multispecific proteins include Fc-fusion proteins, albumin-fusion proteins, bispecific antibodies, and other formats in which multiple protein domains are linked together into a single-chain.

Many multispecific proteins have already been FDA-approved across various diseases. Fc-fusion proteins are used to treat autoimmune diseases (alefacept, abatacept, and etanercept), macular degeneration (aflibercept), and hemophilia (efmoroctocog alfa).^99^, ^100^, ^101^, ^102^, ^103^, ^104^ Albumin-fusion proteins have been used to treat type 2 diabetes (albiglutide) and hemophilia B (albutrepenonacog alfa and albumin-fused coagulation factor IX); other albumin-fused recombinant coagulation factors are being investigated.^105^, ^106^, ^107^, ^108^ Bispecific antibodies can be designed to target multiple receptors or pathways on the same cell (amivantamab and faricimab) or to bring 2 different cell types into close proximity, improving their ability to target disease sites with greater precision, particularly to bring together T cells and cancer cells (blinatumomab and teclistamab).^99^^,^^109^, ^110^, ^111^, ^112^, ^113^

For CVD, at least 2 such multispecific proteins have been approved thus far. Sotatercept-csrk, approved in 2024, is an activin receptor type IIA-Fc fusion activin signaling inhibitor that treats pulmonary arterial hypertension, which affects the lungs and right side of the heart. Semaglutide (approved for CVD indications in 2020) is a glucagon-like peptide-1 (GLP-1) receptor agonist that has been modified to include a fatty acid chain and shown to reduce cardiovascular risk.^114^^,^^115^

**Fig. 2:**
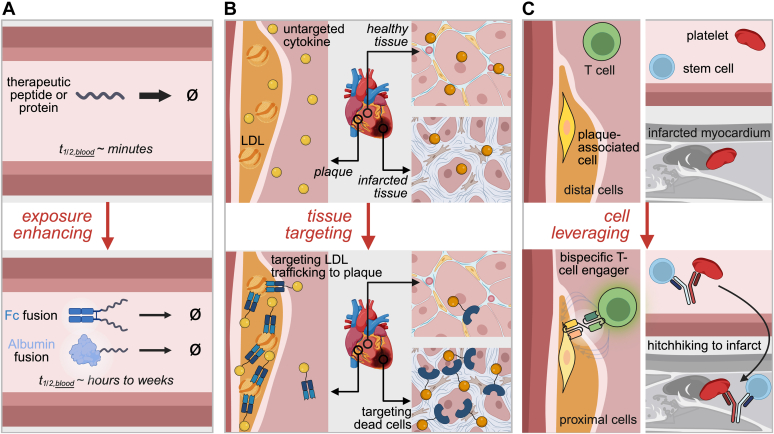
Engineering strategies to optimize protein-based biologics for cardiovascular disease. (A) Enhancing exposure through Fc or albumin fusion extends circulatory half-life, enabling therapeutically relevant systemic exposure of peptides or proteins. (B) Tissue targeting can direct biologics to diseased cardiovascular tissues, such as atherosclerotic plaques or infarcted myocardium, to improve on-target tissue activity and minimize off-target tissue effects. (C) Cell leveraging strategies harness natural cell–cell interactions, such as T cell engagement or platelet hitchhiking, to enhance delivery and localization to cardiovascular disease sites. Created in BioRender (2025), https://BioRender.com/xefeop1.

Expanding this therapeutic class for CVD requires understanding the pharmacokinetic advantages that multispecific architecture provides. Multispecifics can extend half-life to allow more feasible dosing and improve efficacy, enhance tissue targeting to improve safety and efficacy, and leverage cellular interactions that unlock new therapeutic possibilities (Fig. 2).

### 3.2 Exposure enhancing

Fusion of therapeutic proteins to the Fc region (∼50 kDa) of antibodies can extend half-life through size and FcRn-mediated recycling.^116^, ^117^, ^118^, ^119^, ^120^ This approach is particularly valuable for short-lived peptides and proteins that are quickly metabolized or excreted and thus would require frequent dosing as therapeutics. For example, unmodified atrial natriuretic peptides (ANPs), which are natural hormones that aid in blood pressure, and fluid and electrolyte balance, exhibit rapid degradation and clearance, which limits their half-life to 2 to 5 minutes.^121^^,^^122^ However, when modified via an Fc fusion, ANP-Fc prolongs the half-life of the fusion by 2 orders of magnitude through FcRn-mediated recycling compared with unmodified ANP (Fig. 2A).^123^^,^^124^ The dimeric nature of Fc-fusion also promotes avidity, creating a more potent dimeric ANP-Fc compared with monomeric ANP-Fc fusions.^124^

Similar to fusion to Fc, fusion or binding to albumin also achieve half-life extension. Albumin (∼66 kDa) is the most abundant protein in blood plasma and acts as a transporter for fatty acids. It exhibits an extended half-life of 3 weeks in humans because of its size, charge, and ability to be recycled by FcRn (Fig. 2A).^125^ Therapeutic proteins fused to albumin directly or that bind via another protein domain or fatty acid can therefore leverage this natural longevity. For example, AlbuBNP, a B-type natriuretic peptide fused to human serum albumin extends the 3 minute half-life of unmodified B-type natriuretic peptide to 12 to 19 hours with similar levels of bioactivity in preclinical studies.^126^ Rather than direct fusion to albumin, fusion proteins have also hitchhiked off of natural serum albumin to extend half-life. Semaglutide, previously mentioned as an FDA-approved drug to mitigate cardiovascular risk, is a GLP-1 receptor agonist that has been modified to include a fatty acid chain to increase its binding to serum albumin and enhance its resistance to enzymatic degradation; this increased the minute-long half-life of native GLP-1 to 7 days, allowing for a once weekly dosage regime while maintaining its agonistic function.^127^, ^128^, ^129^ Similarly, GAlbudAb, a fusion of GLP-1 and an albumin-binding antibody, has demonstrated extended systemic exposure comparable to semaglutide, as well as improved lipid profiles in a preclinical setting, which could translate into benefits for CVD management, especially in patients with dyslipidemia and heart failure.^130^, ^131^, ^132^, ^133^

### 3.3 Tissue targeting

Beyond half-life extension, multispecific proteins can be engineered to achieve more selective targeting of cardiovascular tissues. This approach enhances both the safety and efficacy of these agents by concentrating activity at diseased sites and away from uninvolved healthy tissues. Targeting can be achieved by employing proteins that bind targets upregulated in diseased states or enriched in diseased tissues. Recent preclinical work highlights several promising examples of targeting. An LDL-targeted IL-10 fusion reduces inflammation in atherosclerotic plaque, which exhibits elevated LDL levels (Fig. 2B).^134^ Stromal cell-derived factor 1-annexin V delivers stromal cell-derived factor 1 to infarcted myocardium via dead-cell binding to protect from ischemic injury (Fig. 2B).^135^ Immunocytokines—cytokines fused to mAbs or their fragments—can also be used for tissue-specific targeting. For example, IL-10 fused to a homodimeric single-chain variable fragment diabody targeting extra domain A—an alternative splice variant of fibronectin—has been shown to target cardiac allograft rejection, as rejection leads to overexpression of extra domain A, but showed no therapeutic benefit.^136^ Because some immunocytokines can retain mAb-like PK, such as extended serum half-life, this format can also result in the same side effects because of the distribution in healthy tissues that limit standalone mAbs.^137^^,^^138^

### 3.4 Cell leveraging

Multispecific proteins can be engineered to leverage natural cells in the body by agonizing or antagonizing multiple pathways on the same cell, or facilitating interactions between different cells. Respectively, the goal is to harness complementary signaling pathways to achieve enhanced or synergistic therapeutic effects, or to promote cellular crosstalk to improve a cell’s ability to target disease sites with greater robustness or precision. Bispecific antibodies exemplify the latter strategy. Among this class, bispecific T cell engagers (BiTEs) represent a particularly promising format. BiTEs are engineered antibodies or antibody fragments with 2 distinct binding sites, 1 targeting a molecule on T cells via anti-CD3, and the other targeting a molecule on a target cell type. BiTEs have shown tremendous promise in oncology by directing immune cells to target cancer cells, and cardiovascular applications are emerging.^139^ For example, preclinical data demonstrates efficacy of an anti-fibroblast activation protein BiTE to treat atherosclerotic coronary artery disease (Fig. 2C).^140^ Another innovative application of bispecifics involves “hitchhiking” on circulating cells for enhanced delivery. A CD34-CD42b bispecific antibody targets hematopoietic stem cells via CD34 and platelets via CD42b, enabling hematopoietic stem cells to piggyback on platelets to reach sites of myocardial infarction where they can contribute to tissue repair (Fig. 2C).^141^ This strategy leverages the natural trafficking patterns of platelets to overcome delivery barriers and achieve targeted cellular therapy.

## 4 Emerging protein engineering approaches for cardiovascular therapeutics

Several engineering strategies are being explored to improve the on-target, on-tissue activity of protein therapeutics, many of which can be applied to CVD therapy. These strategies are generally rooted in either tuning the circulatory half-life or improving the target tissue accumulation. Advances in Fc engineering are one promising approach, such as tailoring Fc variants to have either extended or shortened half-lives via Fc mutations—the triple mutation M252Y/S254T/T256E (YTE) enhances binding to the FcRn to increase half-life, for example.^142^ Another emerging strategy involves optimizing the affinity of binders for their targets. Although high-affinity target binding is often desirable, excessively high affinity can impair tissue penetration and increase off-target tissue exposure. Affinity tuning can involve several methods, including site-directed mutagenesis, chain shuffling, and error-prone polymerase chain reaction.^143^, ^144^, ^145^, ^146^, ^147^ Fine-tuning these parameters, including dosage, can allow for better discrimination between healthy and diseased tissue, minimizing systemic toxicity and improving therapeutic windows.

In addition, nanobodies and other smaller targeting domains, such as those developed to target PCSK9 or vascular cell adhesion molecule 1, also offer a strategy for maintaining high targeting precision while decreasing circulation time and half-life.^148^, ^149^, ^150^, ^151^, ^152^ These smaller biologics provide rapid tissue penetration because of their smaller size and lower off-target toxicity because of their shorter half-life, making them a promising alternative to conventional mAbs in scenarios where prolonged circulation may be detrimental.^148^^,^^153^ Aside from nanobodies, several alternative highly specific, small scaffolds are also being used for strong target binding, such as designed ankyrin repeat proteins, affibodies, and Anticalins.^154^, ^155^, ^156^, ^157^ These scaffolds are being rapidly optimized using high-throughput display technologies (eg, phage, yeast, or ribosome display), alongside computational protein design and deep mutational scanning, which further accelerates the development of high-affinity binders.^158^, ^159^, ^160^, ^161^, ^162^, ^163^, ^164^, ^165^, ^166^, ^167^

Targeting peptides or small proteins (<30 kDa) can also offer fast tissue distribution and rapid clearance from the bloodstream. This effect, alongside targeted binding, can promote tissue retention and on-tissue specificity. For the heart, cardiac-targeting peptide, which was identified via in vivo phage display, demonstrates selective homing to cardiomyocytes with minimal off-tissue uptake in other organs in treated mice.^168^, ^169^, ^170^, ^171^ Also, an ischemia-targeting peptide has been shown to preferentially localize to the ischemic myocardium in myocardial infarction-reperfusion models.^172^, ^173^, ^174^ By leveraging quick redistribution and clearance from the bloodstream, small targeting peptides or proteins fused to a therapeutic cargo can theoretically concentrate in target tissues with negligible systemic exposure and toxicity.^175^

Future development of multispecific proteins for CVD will benefit from the rational incorporation of binding kinetics and pathology-specific proteolytic environments into their design. Low-affinity, high-avidity binding architectures have been shown to enhance specificity via expression-dependent targeting, limiting stable binding to tissues with high target density.^176^ In such systems, individual domains bind weakly (ie, fast off-rates), but their multivalent configuration enables cooperative, high-avidity binding only at disease sites with elevated target expression, thereby minimizing off-target interactions in healthy tissues. Computational models of low-affinity, high-avidity constructs have proven highly effective at predicting binding kinetics in vitro, providing a valuable framework to guide their rational design.^177^, ^178^, ^179^ Although not yet applied to CVD models, these design strategies are particularly promising for targeting CVDs, where the tissue is very heterogeneous.

Another emerging direction is the engineering of protease-responsive fusion proteins (ie, masked therapies), such as those activated by matrix metalloproteinases (MMPs)—in particular, MMP-9, MMP-2, and MMP-14 are upregulated in the context of cardiac remodeling and atherosclerosis.^180^, ^181^, ^182^, ^183^, ^184^, ^185^ MMP-activatable constructs remain latent in circulation and become functional only upon cleavage by MMPs, which can expose a previously hidden effector domain, enabling spatiotemporal control over drug activation and reducing systemic exposure.^186^, ^187^, ^188^ These have been shown to selectively localize to and become activated in disease tissues, such as tumors, where MMP activity is elevated, allowing cytokines, mAbs, and BiTEs to achieve potent therapeutic effects while minimizing systemic toxicity.^189^, ^190^, ^191^ An MMP-activatable anti-vascular cell adhesion molecule 1 construct has been shown to specifically accumulate in aortic plaques in an apolipoprotein E mouse model.^192^

Further, identification of more CVD- and heart-specific proteins, followed by engineering of binders for those targets, would help minimize systemic exposure to improve the safety of these biologics. By leveraging advances in biologic engineering and delivery technology, protein-based biologics have the potential to become powerful tools in treating CVD, offering their ability to modulate half-life of any protein or peptide, combine unique effector domains with efficient targeting domains, or increase disease or tissue specificity with multiple targeting domains.

## 5 Cardiovascular delivery and transport considerations

The PK processes governing mAbs and multispecific proteins take on additional complexity when considering the specialized anatomy, hemodynamics, and tissue composition of the heart and its vessels. Integrating this perspective on cardiovascular transport is essential for engineering protein-based biologics for CVD treatment.

Despite comprising only 0.5% of body mass, the heart receives 5% of cardiac output (about 250–300 mL/min at rest), reflecting its high metabolic demands.^193^ This high perfusion, combined with variable hemodynamic pressures across cardiac chambers, ranging from 4 to 5 mmHg in the right atrium to 15 to 120 mmHg in the left ventricle in a healthy heart, creates dynamic pressure gradients that directly influence a drug’s transport and penetration into the myocardium.^33^^,^^194^^,^^195^

Biologics move in and out of the myocardium from the blood by either crossing endothelial cells, which comprise the barrier between vessels and tissues, or traversing between them. The size and charge of proteins generally precludes their ability to traverse across a cell, unless facilitated by an active transport process such as the FcRn-mediated transcytosis used by antibodies.^196^, ^197^, ^198^, ^199^ The alternative paracellular path depends on the permeability of the endothelial barrier in that tissue. The heart, lined with continuous nonfenestrated endothelium, creates a more restrictive barrier to passive transport compared with other tissues (eg, liver, kidney, spleen, and bone marrow) and is typically permissive to water and solutes with molecular radii <3 nm, which roughly corresponds to proteins between 40 and 70 kDa.^200^ Furthermore, the thickness of the endothelial cell varies from <0.1 μm in capillaries and veins to 1 *μ*m in the aorta.^201^ These transport considerations have important implications for biologic design: there may be a size advantage for smaller biologics to reach cardiovascular tissues.

Importantly, this endothelial barrier is not static. Cardiovascular pathologies fundamentally alter its permeability in ways that can dramatically change biologic transport. Inflammation, ischemia, and fibrosis can disrupt endothelial integrity and increase vascular permeability, thereby potentially expanding the size-cut off for passive paracellular transport and enhancing biologics' extravasation into affected tissues.^202^, ^203^, ^204^

However, these same pathologic processes can simultaneously impair biologic delivery and distribution through counteracting mechanisms. High blood pressure and/or impaired lymphatic drainage, common in patients with heart failure, leads to fluid leak and accumulation increasing the interstitial pressure, which ultimately opposes drug penetration in the myocardium.^205^, ^206^, ^207^ Fibrotic remodeling creates a dense extracellular matrix that shrinks the average pore size of the matrix (10–50 *μ*m in healthy tissue) and increases tissue stiffness and charge.^208^, ^209^, ^210^, ^211^ This physical barrier impedes diffusive transport of macromolecules within the tissue.^16^^,^^212^^,^^213^ Protein therapeutics span a wide continuum of sizes (12 kDa nanobodies to >200 kDa multispecific antibodies), straddling the transition where cardiovascular transport mechanisms diverge. As such, larger biologics may become trapped at the endothelial interface or within fibrotic scar, whereas smaller formats can more easily diffuse through denser matrix. Dense fibrosis also correlates with reduced vascularity and perfusion (microvascular rarefaction) diminishing convective delivery.^214^, ^215^, ^216^, ^217^ Extensive calcification (calcified plaque) can also introduce rigid, virtually impermeable regions within plaques, which have been shown to limit intravascular drug delivery to human peripheral arteries.^218^, ^219^, ^220^ Additionally, reduced perfusion due to coronary artery disease or ischemic conditions may itself prevent adequate delivery of biologics to affected tissues.^221^, ^222^, ^223^, ^224^ Although these barriers are universal, their practical impact scales with molecular size, charge, and affinity of the therapeutic. Thus, although smaller scaffold sizes, as in the case of protein therapeutics, can partly bypass steric obstruction, all therapeutics still face compounded hurdles in fibrotic or calcified myocardium.

The heart’s structural complexity further complicates biologic delivery. Distinct anatomical layers (endocardium, myocardium, and epicardium) as well as 4 chambers (left and right atria and ventricles) each present unique tissue microenvironments, varying in cellular composition, vascular density, and extracellular matrix architecture. These regional differences influence biologic transport and choice of targets that can be leveraged for tissue-specific delivery.^9^^,^^212^^,^^225^, ^226^, ^227^, ^228^, ^229^, ^230^, ^231^, ^232^

Pathophysiology of CVD further alters these tissue microenvironments, creating additional variables that must be considered in therapeutic design.^16^^,^^212^^,^^213^ Increased blood pressure, fibrotic tissue from ischemic cardiomyopathy or cardiac amyloidosis, or limited perfusion from peripheral artery disease can all complicate biologic delivery.^221^, ^222^, ^223^^,^^233^ For example, because the pressure levels in the heart are determined by vascular hydrostatic forces and interstitial fluid removal—with excess fluids drained by the lymphatic system—conditions such as heart failure that impair lymphatic function can lead to fluid accumulation and increase interstitial pressure, which can hinder drug delivery.^205^, ^206^, ^207^ Extracellular matrix and cell composition also change in disease conditions.^16^^,^^212^^,^^213^

Additionally, comorbidities commonly seen in patients with CVD—including diabetes, chronic kidney disease, and obesity—can further complicate the PK/PD profiles of biologics.^234^ For example, diabetes-induced microvascular damage impairs tissue perfusion and endothelial transport, reducing biologic delivery to cardiovascular tissues.^235^, ^236^, ^237^ Similarly, renal or hepatic dysfunction may affect systemic clearance and metabolism of biologics, particularly for those that rely on nonspecific catabolic pathways.^33^^,^^238^^,^^239^ Polypharmacy is often necessary to manage these multiple comorbidities. However, coadministration of biologics with small molecules or other biologics can introduce drug–drug interactions that influence PK/PD.^240^^,^^241^ For instance, statins have been shown to upregulate PCSK9 expression, thereby altering the PK of anti-PCSK9 antibodies such as alirocumab and shifting their clearance profile toward TMDD mechanisms.^43^^,^^242^^,^^243^ Understanding and anticipating such interactions is essential for dosing optimization and reducing variability in therapeutic response among patients receiving multiple cardiovascular agents.

As protein-based biologics advance into cardiovascular applications, understanding their PK profiles specifically in the context of cardiovascular physiology and pathology is crucial for their successful translation.

## 6 Conclusions and future perspectives

Biologics represent a rapidly expanding and versatile class of therapeutics with significant potential to reshape CVD treatment. The success of protein-based biologics—particularly mAbs and multispecific proteins—in other diseases has been driven by engineered control of PK/PD. Dissecting how features such as FcRn-mediated recycling, TMDD, and immunogenicity influence biologic exposure and efficacy elucidate strategies for optimizing therapeutic outcomes in CVD. Multispecific proteins exploit these same PK principles to control their PD profiles by fusing proteins of different targets together. These designs can allow multispecific proteins to extend half-life, improve on-target on-tissue localization, or leverage natural cells in the body to agonize or antagonize multiple targets on the same cell simultaneously or bring 2 cells together so they interact with one another. Although this review highlights representative examples of these advances and design principles, there is a vast number of ongoing preclinical and clinical studies that continue to inform the field and cannot be exhaustively summarized here.

An emerging class of biologics includes intracellular therapies—such as small interfering RNA, antisense oligonucleotides, mRNA, and CRISPR-associated protein 9—that enable potent gene-silencing, -enhancing, or -editing effects.^244^^,^^245^ To achieve targeted delivery and efficient cellular uptake, these agents can rely on protein-based carriers, including protein-coated lipid nanoparticles and extracellular vesicles.^246^, ^247^, ^248^, ^249^, ^250^, ^251^, ^252^, ^253^, ^254^, ^255^, ^256^ However, their therapeutic activity depends on successful trafficking to specific subcellular compartments (eg, the cytosol or nucleus), introducing a new layer of PK/PD complexity.^247^^,^^248^^,^^257^^,^^258^ As with any drug modality, these intracellular biologics require careful consideration of these PK/PD factors to guide their design.

To further improve the development of biologic therapies for CVD, innovations in PK modeling are critical. In silico models—including physiologically based PK models and quantitative systems pharmacology—are offering new tools to predict and optimize cardiac-specific biodistribution and therapeutic outcomes.^259^, ^260^, ^261^, ^262^ For example, a 4-compartment physiologically based PK heart model has been developed that incorporates distinct regions of the myocardium (epicardium, midmyocardium, endocardium, and pericardial fluid) and accounts for cardiac metabolism, enabling more precise estimation of local drug concentrations and metabolic clearance in the heart.^259^ Complementary quantitative systems pharmacology approaches are being used to simulate complex physiologic processes underlying heart failure and drug response, and to evaluate interventions in virtual patient populations.^260^ Integration of machine learning with quantitative systems pharmacology has further enhanced the ability to personalize these models to refine dosing strategies and safety assessments, as well as support mechanistic hypotheses and regulatory decision-making in CVD drug development.^261^^,^^262^ These models allow for more precise predictions of how biologics will behave in the complex and dynamic environment of the heart, thereby aiding in the design of more effective treatments and enhancing personalized therapy. Additionally, molecular dynamics simulations and artificial intelligence platforms—such as AlphaFold, Rosetta, BindCraft, EvoBind, and SaLT&PepPr—are increasingly used to guide biologic design by predicting structure from sequence and modeling how structural features or motifs govern target binding, stability, and other properties relevant to therapeutic function.^224^^,^^263^, ^264^, ^265^, ^266^, ^267^, ^268^, ^269^, ^270^, ^271^ These platforms have the potential to accelerate biologic design and development, but still require user-defined input—such as the desired target, scaffold shape, and size—to ensure that candidates are optimized not just for binding, but for therapeutic efficacy with favorable PK/PD properties.

Looking to the future, protein-based biologics offer an unprecedented opportunity to reshape cardiovascular therapy by enabling precise, durable modulation of complex pathophysiologic processes, ranging from inflammation to fibrosis. Realizing this potential will require a deliberate, PK-informed approach to engineering that accounts for the heart’s unique anatomical, cellular, and physiologic barriers. As biologics grow more sophisticated and new therapeutic modalities emerge, a deep understanding of PK/PD is essential to guide their development. Innovative experimental protein engineering techniques and in silico modeling tools will help predict, optimize, and tailor biologic behavior in cardiac tissue, where unique anatomical and physiologic constraints can profoundly impact drug distribution and action. PK/PD-informed design of protein therapeutics could fundamentally shift how we treat CVD, enabling highly targeted, durable interventions that reshape disease trajectories and meaningfully improve patient outcomes.

## Conflict of interest

Noor Momin is an inventor on US Provisional Patent application nos. 63/501,286 and 63/525,135 regarding antibody-based treatments for atrial fibrillation and no. 63/719,486 regarding fusion protein treatments for cardiovascular disease. Emily Lin declares no conflict of interest.
